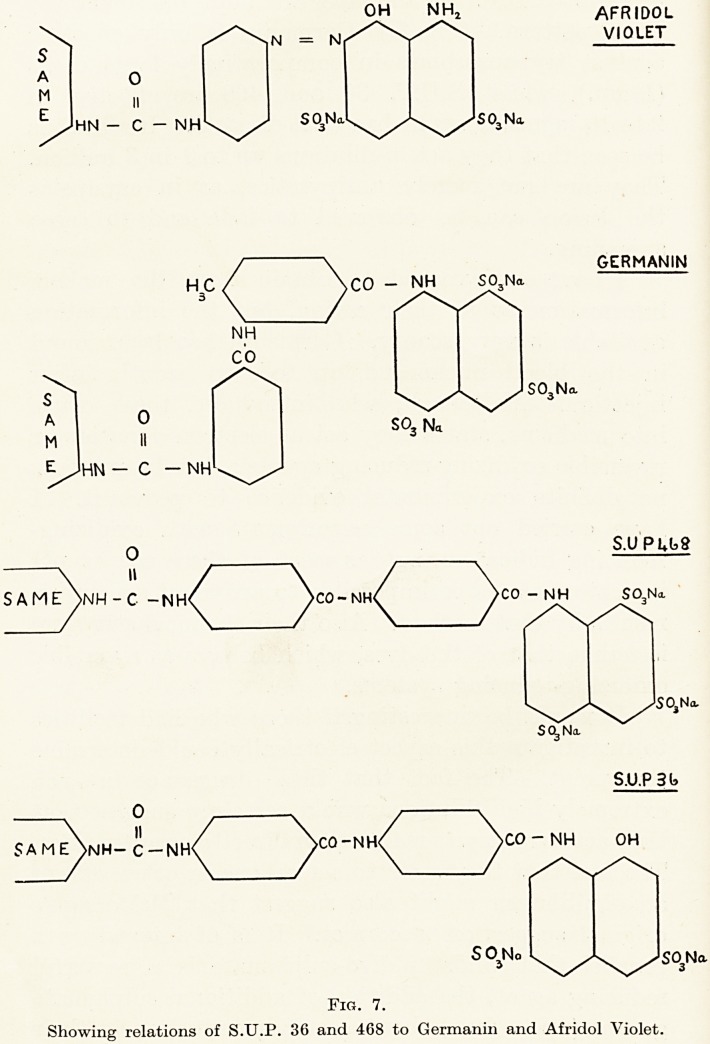# Ultra-Microscopic Examination of the Blood-Serum in Disease
*A paper read at a Meeting of the Bristol Medico-Chirurgical Society, with cinematograph demonstration, on Wednesday, 8th January, 1936.


**Published:** 1936

**Authors:** B. A. Peters

**Affiliations:** Medical Superintendent, Ham Green Hospital


### ULTRA-MICROSCOPIC EXAMINATION OF THE BLOOD-SERUM IN DISEASE.*

BY

B. A. PETERS, M.D., D.P.H.,

*Medical Superintendent, Ham Green Hospital.*

---

DURING the last six years we have examined, by means of the dark ground condenser or ultra-microscope, many hundreds of specimens of blood-serum in health and disease. The limit of resolution of an ordinary microscope is 0·1 $\mu$. With the ultra-microscope particles of 5–10 $\mu\mu$ are rendered visible if they have a different refractive index from that of the medium in which they are suspended. By this technique the serum is seen to contain numerous particles in active Brownian movement.

My attention was first drawn to this method of investigation by the work of J. E. R. McDonagh.[1] He pointed out that there were striking differences in the appearances of the serum in disease from those in health. He also averred that, after the administration of certain drugs, the serum appearance returned to normal with improvement in the clinical condition. On the strength of these observations he constructed what is practically a new system of medicine, with the unfortunate result that the truth and accuracy of his observations have been buried beneath a mass

* A paper read at a Meeting of the Bristol Medico-Chirurgical Society, with cinematograph demonstration, on Wednesday, 8th January, 1936.

of speculation which has received little acceptance either from chemists or physicians.

I have attempted to examine the position afresh, free as far as possible from preconceived ideas, at the same time consulting widely the literature of colloidal and bio-chemistry as far as it is likely to throw any light on the interpretation of the phenomena observed. One of the great drawbacks in arousing any interest in these observations has been the difficulty of demonstrating the phenomena to others, as the specimens must be observed within a few hours after taking the blood, and cannot be preserved unchanged. As the objects are in active movement their demonstration by still photography is unconvincing.* But as the result of many hundreds of observations in different acute infections I am convinced that certain appearances are related to certain clinical conditions.

The specimens are taken thus: About 0·5 c.c. of capillary blood is taken from the ear and allowed to stand in the incubator for four hours in a small test-tube. I think capillary blood shows the changes best, as there the blood is in most intimate contact with the tissues, and any large particles are most likely to be temporarily arrested there. Blood examined outside the body by any method is necessarily in an altered state, but if each specimen is examined under the same conditions its appearance should bear some relation to its condition *in vivo*.

The first specimen is whole normal blood photographed within two minutes of collection before it has time to clot. (Fig. 1.) You will observe, in channels

* This research has been carried out by means of micro-cinematograph; a film was shown to illustrate the paper. The author wishes to acknowledge his indebtedness to the Sir Halley Stewart Trust for a grant towards this piece of work, and to Mr. K. Dunscombe for his assistance on the technical side.

PLATE II.

Fig. 1.
Whole fresh blood, showing numerous particles in plasma.

Fig. 2.
Normal serum, showing two sorts of particles.

between masses of aggregated corpuscles, the drifting plasma, which shows numerous particles in extremely active Brownian movement. The next specimen is a photograph of serum which has exuded from the clot after four hours in the incubator. (Fig. 2.) You will observe that the particles are similar to those observed in fresh blood, demonstrating that the particles in serum are neither artefacts nor products of cell disintegration, but are present as such *in vivo*.

The normal particles are of two kinds, some bright, and some greyish which do not photograph so well. There are slight individual varieties in healthy subjects in the number and apparent size of the particles, but the variations are not great. If the blood is taken too soon after a fatty meal numerous sticky, comparatively large, globules of fat may be seen also. I would draw your attention to the macroscopic appearance of the clot of normal blood after four hours at 98·4° F. The clot is adherent to the top of the tube and is well retracted, leaving clear serum round the edges. (Fig. 6*a*.)

At this stage one would naturally inquire as to the nature or composition of these particles, as obviously these countless myriads are not placed in the blood by nature to amuse the photographer. McDonagh called them protein particles. I do not think that this view is tenable, because the refractive index of the heavily-hydrated protein is so nearly that of the water in which they are suspended that protein particles would not show by this method of investigation. If alcohol is run in under one of our films an enormous increase of particles occurs with precipitation of sheets of coagulum, showing that at least most of the protein is not visible unless dehydrated and precipitated. A calculation based on the osmotic pressure of plasma protein shows that, to produce this pressure, the particles should be 2,000,000 times as numerous as they are. Also, the red-blood corpuscles are optically empty, showing that the protein hæmoglobin is not visible. The blood contains appreciable amounts of sterols such as cholesterol (either free or combined with fatty acids), of fatty acids, and of lipins such as lecithin and cephalin. An emulsion of lecithin and cholesterol is very stable, and resembles in its optical properties the particles seen in the serum. The fat globules seen in serum are much larger and tend to adhere to corpuscles and fibrin threads and become strung into sausage-like strings.

Some lecithin and cholesterol estimations in blood-serum, kindly carried out by Professor Walker Hall and his staff in the Department of Preventive Medicine, show some correlation with the number of particles observed in different sera ; but the observed differences in the numbers of the particles are much greater than the differences in amounts of lecithin and cholesterol found in the serum. These results would tend to negative the idea of these particles being lipins or sterols ; but it is possible that in some cases they might exist in too fine a state of subdivision to be visible even by this method. It would require an aggregation of many molecules of any of these substances to render them visible, as the limits of visibility are probably 5–10 $\mu\mu$. They may even be particles derived from leucocytes, but at the moment most of the evidence in my possession suggests a lipoid nature.

In disease some striking departures from the normal picture can be observed, and in some cases the protein particles literally enter the picture. The differences observed are in the numbers and apparent size of the particles normally present or the state of dispersion, and in certain cases the appearance of a new form of particle.

To understand the factors involved in these phenomena I must ask you to consider some accepted facts in colloidal chemistry. The plasma is a solution of electrolytes molecularly dispersed in solution, but in addition holds the proteins, fats and lipins. According to Svedberg's work,[2] the proteins are enormous molecules with molecular weights of some multiple of 34,500. Apparently serum-globulin and serum-albumin exist as single molecules of protein; whilst fibrinogen, euglobulin and pseudo-globulin consist of aggregations of variable numbers of molecules. Owing to the enormous sizes of these molecules they fall within the dimensions of the colloid realm, which is taken as 1 $\mu\mu$ to 100 $\mu\mu$. They may be regarded as globules surrounded by, and floating in, hundreds of the very much smaller molecules of water and its dissolved salts. The fats certainly, and the lipins probably, exist as floating masses of molecules of these substances.

These colloid particles are too large to be in quite the same state as dissolved electrolytes. The Brownian movement which they exhibit is due to their being small enough to jump about under the irregular bombardment of other molecules which counteracts gravitational forces and keeps them from settling out except after a very long time. The force of surface tension tends to draw them together into larger masses, but this tendency is opposed owing to every particle carrying a similar electric charge which tends to repel one from the other. The sign of the electric charge, whether it is + or —, depends on the pH

of the solution in which they are suspended. At a certain pH they carry no charge, and at this point (the iso-electric point) they are unstable and tend to form larger aggregates, which separate out. At the reaction of the blood (pH = 7·4) they carry a negative charge. The colloids of the body are termed hydrophilic, *i.e.* they tend to bind or to lose water according to the pH of the blood and also according to the concentration of the salts in which they are floating. This property also renders the suspension very stable compared with metallic colloids, which are at once precipitated if the electric charge they carry is discharged by an ion in solution having the opposite charge.

Any factor, therefore, which causes these particles of the blood to lose their electric charge, to increase their surface tension or to dehydrate them, will tend to cause them to form larger aggregates and to flocculate out. We know that under certain conditions the body fluids can cause agglutination of typhoid bacilli or cause the flocculation of foreign protein—typical colloidal reactions—whereas under other circumstances these foreign particles can be broken down into smaller aggregates with loss of their special properties (that is, proteolysis or bacteriolysis). As the body can do this to the invader, it does not seem to be a wild flight of the imagination to consider that the invader may have similar effects on the body colloids. I think that the specimens seem to show there is evidence of these occurrences in the blood during the acute attack.

The next specimen shown is serum from a case with a chronic infected antrum (a streptococcal infection). (Fig. 3.) You will observe that the particles are in comparatively large masses, and as a consequence the Brownian movement is sluggish, with a tendency for the particles to settle out. Such appearances are characteristic of chronic infections with streptococci or staphylococci. The patients are comparatively resistant to severe acute infections. From observations of the sera of several hundred cases of erysipelas, we find that all the mild cases show such an appearance. The blood-sugar is generally normal. In a case of erysipelas with such a serum one or two doses of 0·125 contramine, 10 mgms. manganese butyrate or 0·002 gm. S.U.P. 468, or anti-toxic serum, will cause an immediate cessation of the spread of the disease in the skin with a drop of temperature to normal in twelve hours. The same drugs will at once cure furunculosis if the blood shows this picture. In more chronic cases colloidal sulphur will act efficiently.

In all cases with blood of this nature the general health is generally poor, but improves strikingly on this treatment. These drugs all cause the blood rapidly to resume the normal picture. Thyroid medication also has the same effect in some degree, also glucose. You will realize that the breaking up of these large masses into smaller particles increases the surface area of the material and produces a larger reactive surface. The work of Walsh and Frazer[3] is highly relevant to this discussion. They showed that emulsions of olive oil had the power of neutralizing a large amount of toxin if mixed prior to injection. They pointed out that no fixation of toxin occurred unless the emulsion was in a very fine state of dispersion. They claimed considerable therapeutic value for the intravenous administration of this emulsion in various acute infections.

We suggest that the effect of the toxin of these organisms is to reduce the electric charge of the particles (*i.e.* it acts as an oxidizing agent in the sense used in modern chemistry of depriving the particles of electrons). Hence they form larger aggregates. The effect of the drugs mentioned is stated to be a reducing one—*i.e.* they give up hydrogen or electrons to the particles, hence these tend to fly apart into smaller masses, thus presenting a larger reactive surface capable of absorbing the foreign toxin. The macroscopic appearance of the clot is similar to that of normal blood. (Fig. 6*a*.)

The next specimen is from a case of malignant streptococcal infection where the patient is being overwhelmed. (Fig. 4.) You will observe that the blood is almost entirely empty of particles. In less severe cases the larger particles are replaced by numerous extremely small particles which will not photograph well. I think this is an earlier stage of the condition demonstrated, and that here the particles have been either broken up into particles too small to be visible or else have been precipitated out in the capillaries. This appears to be the exact opposite of the other specimen. Such cases are made much worse by the methods used for the former group, and anti-streptococcal serum may be entirely useless. They are best treated with a low carbohydrate diet (the blood-sugar is generally raised above the normal), antitoxic serum, small doses of insulin and S.U.P. 36 ; but one must confess that most forms of treatment at our disposal at present are usually very ineffective.

On the hypothesis that the missing particles may be cholesterol or lecithin or some of their compounds, I treated four severe cases of erysipelas with subcutaneous injections of 1·0 per cent. cholesterol

FIG 3.

Serum in chronic streptococcal infection. Particles reduced in number and increased in size. (Condensation or aggregation.)

FIG. 4.

Serum from malignant streptococcal infection. Only four small particles in whole field. Circles are red-blood corpuscles.

PLATE IV.

Fig. 5.

Serum in acute tuberculosis. Particles very numerous, flocculating out into sheets of precipitate (gelation). The three bright circles only are red-blood corpuscles.

c a b

Fig. 6.

Macroscopic appearance of clot after four hours in incubator.

*a.* Normal blood and blood showing condensation of particles.

*b.* Blood from case where particles are reduced or nearly absent.

*c.* Blood from case showing gelation.

and 0·1 per cent. lecithin emulsion (supplied by British Colloids, Limited). These compounds produced localized indolent necrosis of the tissues with pus containing large amounts of fat globules. In these four cases improvement followed the injections. When given intravenously in other cases they produced no effect, good or bad. As the bio-chemists state that cholesterol and lecithin mobilize the fats of the tissues in the form of esters, and as the appearance of the pus suggested some such phenomena, I had made (by Crookes) emulsions of cholesteryl oleate, which I am trying at present; but so far I cannot say what the effects are going to be, as the number of malignant cases encountered is very small.

When one considers that streptococci have many forms of attack, *e.g.* by the production of a rash-producing exotoxin or a hæmolytic toxin, and also have the power of invading the tissues readily, it is not surprising that they may produce different types of reaction in the serum according to the former state of the patient and the strain of organism. It is probably the multiplicity of its weapons which makes it such a dangerous and intractable invader. In bloods of this type there is little or no retraction of the clot and very little separation of serum. (Fig. 6*b*.)

The next specimen of serum is from a case of acute tuberculosis. (Fig. 5.) The same appearance is often seen in typhoid, especially if phlebitis is present, in other forms of phlebitis, some cases of acute streptococcal infections, and cases of broncho-pneumonia or post-operative pneumonia. It is only seen in patients who are acutely ill and febrile. It is very rarely seen in scarlet fever, and I have not yet encountered it in diphtheria nor in health. You will observe that the serum contains myriads of particles which have a tendency to settle out into aggregated masses. Small lymphocytes are usually entangled in the precipitated masses, suggesting that they have some part in the process. This appearance is seldom universal over the whole field : parts of the field may show the usual particles. The more severe the case the larger are these patches in the preparation. I think in these cases the proteins are involved being dehydrated and precipitated.

In recent cases of tuberculosis with much fever this phenomenon is most pronounced in summer, when high temperatures and sweating tend to dehydrate the patient. Over-exposure to sun has the same effect. This may explain why excessive sun-bathing is bad for the sufferer from acute tuberculosis, a fact we have known for many years. It may also explain why diseases such as influenza, which also cause this phenomenon, aggravate tuberculosis. High carbohydrate diet tends to aggravate it, while high protein and fat diet counteracts it. Anything therefore which tends to produce further dehydration aggravates this condition. It is interesting to note that exposure of serum to ultra-violet light or to X-rays produces the same appearance *in vitro* (Abramson). [4] A similar appearance can be produced by running in alcohol under the slide (which we know causes precipitation by dehydration of the protein), or by half saturation with ammonium sulphate.

It is not a delayed coagulation, for the coagulation of fibrin occurs in a meshwork of very fine needles or fibrils. In this condition the serum proteins (albumin or globulin) are so unstable that the physico-chemical changes of coagulation seem to cause localized areas of secondary protein flocculation. This probably occurs *in vivo* in the lung in post-operative or broncho-pneumonia. It is probably a reversible flocculation, such as occurs when globulin is half saturated with $NH_4$ $SO_4$, as cases of post-typhoid phlebitis treated with S.U.P. 36 clear up in as many days as they formerly did in weeks.

Cases of acute infection (except tuberculosis) showing this picture respond amazingly well to a couple of doses of 0·010 gm. S.U.P. 36. The temperature comes down in a couple of days with disappearance of symptoms and commencing resolution of the lesion. The broncho-pneumonia of measles and whooping-cough in children is rapidly benefited by this drug, as I have found in very numerous cases, unless the disease is in a very late stage. Tuberculosis responds to some extent, but nothing like so effectively as the other diseases mentioned. Patients whose blood shows this picture are made worse by S.U.P. 468 or by contramine. Their clot is loose and tends to sink to the bottom, expelling a large amount of serum. (Fig. 6*c*.)

The specimens I have shown you are extreme examples of different types of sera: but, as occurs in all biological phenomena, almost infinite gradations are seen from one stage to the other. Nor is the line of treatment suggested infallible, as even if the defensive forces are deployed to the best advantage the invader may be able to overcome them. However, after six years' trial I can affirm that this line gives results far superior to any other tried in certain conditions.

I realize that these observations are only a fragment of the complex phenomena of immunity, but I think they are a fundamental fragment. I cannot even offer you a satisfactory hypothesis which covers all the phenomena observed, but it would appear that the lipoid fraction of the serum is a non-specific absorbent or adsorbent of exotoxins. The special affinity of exotoxins, such as those of diphtheria, tetanus or botulism for the lipoids of the central nervous system has long been known. I think my observations show that the quantity and physical state of these substances affect their functions, as Walsh and Frazer showed with their oil emulsions. I have no definite evidence of what is happening to the proteins in these infections, but the fact, as previously recorded, that dehydrating agents (such as alkalies, hypertonic intravenous dextrose and thyroid) are of great value in diphtheria suggests that they are over-hydrated in exotoxic infections. The study of the intake and output of fluids during the first fourteen days of severe diphtheria proves that water is retained in the early stages ; whilst the fact that there is no obvious œdema suggests that the excess water is bound to the protein and not free. These diseases also show a reduced alkali reserve. In the endotoxic group of infections the phenomena suggest that the lipoid fraction is less obviously affected ; but here the proteins appear to be attacked and dehydrated, so that what benefits one type of disease harms the other. Here the alkali reserve is not depleted.

Before concluding I should like to say a few words about the symmetrical urea products to which I have referred. They are of honourable ancestry and decently connected. In attempting to discover a drug with an elective affinity for trypanosomes, Ehrlich discovered that Afridol Violet had some effect on them. After long trial for better trypanocides, Bayer's synthesized Germanin or Bayer 205. You will see that S.U.P. 36 and 468 are built on much the same pattern. (Fig. 7.) Germanin is only effective against trypansosomes in comparatively large doses (1 gm.), whilst S.U.P. 36 and 468 are effective in 2 to 10 mgms. doses in bacterial diseases. It will thus be seen that they act in dilutions up to 1 in 3 million. They are not merely antipyretics, as in erysipelas the lesion can be observed to fade and to cease spreading.

I have endeavoured to obtain from the makers information as to their action, but the information available is very scanty. Germanin has been found in the blood unchanged up to two months after injection. McDonagh, who introduced these drugs into medicine, states they act as electron donators or reversible oxidizing-reducing agents : but he presents no definite experimental evidence to prove it. I have carried out some experiments with oxidizing-reducing indicators, but as most of these act as pH indicators also it is impossible to arrive at conclusive results by that method. Also their chemical structure is unlike that of the dyes, which behave as reversible oxidizing-reducing systems.

It would be interesting if those who had facilities to investigate this aspect electrically could determine this point. The fact that these drugs act in such extremely high dilutions would certainly suggest that they act as catalysts rather than directly as antiseptics. The fact that they seem to act on one or other side of an equilibrium might also suggest that McDonagh's original suggestion is correct. It is of interest as a parallel that whilst indigo-sulphonate is a powerful reducing agent, the addition of additional sulphonate groups (up to four) shows a steady reduction in their reducing power on the addition of each sulphonate

FIG. 7.
Showing relations of S.U.P. 36 and 468 to Germanin and Afridol Violet.

group. I commend to the notice of pharmacologists and biochemists these compounds for further study, as they seem to present a clue to unravel some of the complexities of immunity phenomena.

I am afraid you will consider my observations too academic to be of much practical value to you. However, the application of this hypothesis to the treatment of malignant diphtheria has given such definite results that it can be applied without troubling to do blood examinations. I have already dealt fully with this method on former occasions,[5] but I show you the results of our experience with the hospital figures for thirty years. (See Table.) I do not claim the very low death-rate in the last six years is entirely due to these new methods. The profession is more on the alert in suspecting possible diphtheria than twenty-five years ago, so that there is a tendency for the milder cases to be recognized which formerly escaped notice. This tends to reduce the total death-rate, and epidemic virulence waxes and wanes. However, when one compares the severe cases only one finds a reduction of 43 per cent. compared with my former experience.

In the broncho-pneumonia of measles and whooping-cough of recent years the administration of 5 mgms. of S.U.P. 36 and its repetition in forty-eight hours has a strikingly beneficial result in this dangerous complication if given early enough. A third dose may sometimes be necessary. This can safely be employed without blood examination. In puerperal infections the blood examinations so far done in a comparatively small group of cases seem to show that S.U.P. 36 and antitoxic anti-streptococcal serum are indicated, and our clinical results so far seem to confirm this if the condition is not too far advanced.

DIPHTHERIA CASES—HAM GREEN HOSPITAL.

| Years. | Total discharges. | Died. | Death-rate per cent. |
|---|---|---|---|
| 1905–09 .. .. .. | 1,956 | 109 | 5·5 |
| 1910–14 .. .. .. | 1,852 | 82 | 4·4 |
| 1915–19 .. .. .. | 1,684 | 90 | 5·4 |
| 1920–24 .. .. .. | 3,439 | 200 | 7·6 |
| 1925–29 .. .. .. | 3,131 | 179 | 5·7 |
| 1930–35 (6 years) .. .. | 4,533 | 125 | 2·7 |

SEVERE DIPHTHERIA CASES ONLY.

| Years. | No. of severe cases. | Per cent. of total discharges. | Death-rate on severe cases only. | |
|---|---|---|---|---|
| 1928–29 (2 years) .. | 243 | 19 | 27 7 | |
| 1930–31 (2 years) .. | 364 | 18 | 14·4 | 16·1 |
| 1932 .. .. .. | 108 | 22·5 | 17·0 | |
| 1933 .. .. .. | 95 | 16·4 | 23·0 | |
| 1934 .. .. .. | 82 | 12 | 18·0 | |
| 1935 .. .. .. | 83 | 11 | 12·0 | |

In phlebitis in any form S.U.P. 36 will give excellent results. In chronic furunculosis as opposed to recent furunculosis contramine intramuscularly and colloidal sulphur by mouth appears to be always indicated. In streptococcal infections generally more care must be used, as here the use of the wrong preparation may be very harmful.

The macroscopic examination of the clot agrees generally with the microscopic appearance. If the appearance of the clot after four hours in the incubator (or six hours in a warm room) is as Fig. 6*a*,

S.U.P. 468, alkalies, thyroid and iodine and plenty of glucose by mouth is indicated. If the appearance is as 6*b* or 6*c*, S.U.P. 36 plus antitoxic serum, a high protein and fat diet and an acid mixture such as Mist. Ferri. Perchlor. is indicated. If the condition does not show some response within twenty-four hours you may know you are on the wrong track and your procedure should be revised. These preparations do not show the toxic effects of metallic compounds.

Our experience suggests that in streptococcal infections the parenteral injection of foreign proteins (to produce protein shock) and of powerful antiseptics is to be strongly deprecated, as they appear to produce profound colloidal changes which *may* be beneficial, but if not, may completely wreck the patient's resistance. I think the phenomena which we have shown you are concerned with non-specific immunity, a subject much neglected compared with the study which has been given to specific reactions. And yet our daily work illustrates how important this non-specific factor must be. We all know that if an infectious disease strikes a family some members may escape entirely, some may be gravely affected, and some very slightly affected. The most striking example is probably the difference between an attack of measles in a healthy, well-nourished child and an ill-fed slum dweller. It has been suggested that vitamin A is concerned in this, but so far the experimental evidence on this point is not very definite. My work suggests that some other fatty or lipoid constituent may be directly concerned.

It is in the hope that further investigations may be carried out on this side of immunity,

D

which to my mind is a very important one, that I have ventured to occupy your time by demonstrating this unfinished but, I hope, not "silly symphony."

REFERENCES.

[1] J. E. R. McDonagh, *Nature of Disease*, London, 1924–27.

[2] Svedberg, *Kolloid—z*, 1930, li. 10.

[3] W. C. Walsh and A. C. Frazer, *Brit. Med. Jour.*, March 10th, 1934, i. 424.

[4] H. A. Abramson, *Electrokinetic Phenomena*, New York, 1934 p. 182.

[5] B. A. Peters, *Medical Officer*, January 24th, 1931; *Brit. Med. Jour.*, 1935, i. 585.

## Figures and Tables

**Fig. 1. f1:**
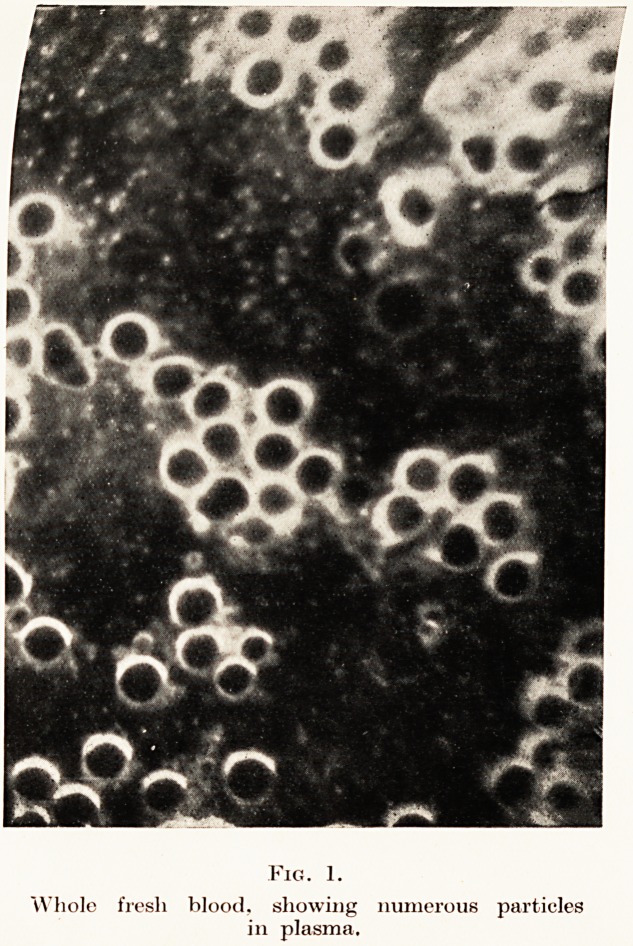


**Fig. 2. f2:**
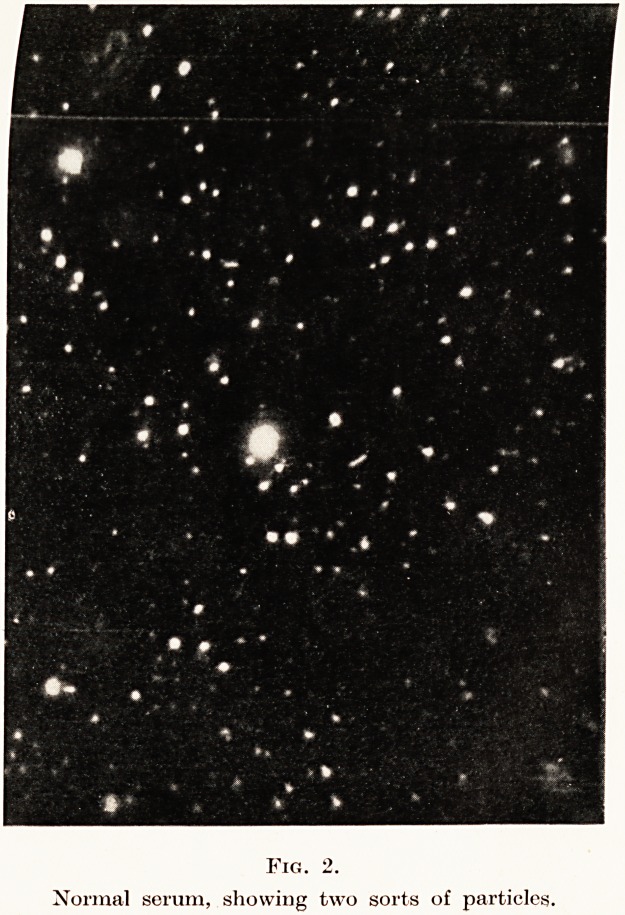


**Fig 3. f3:**
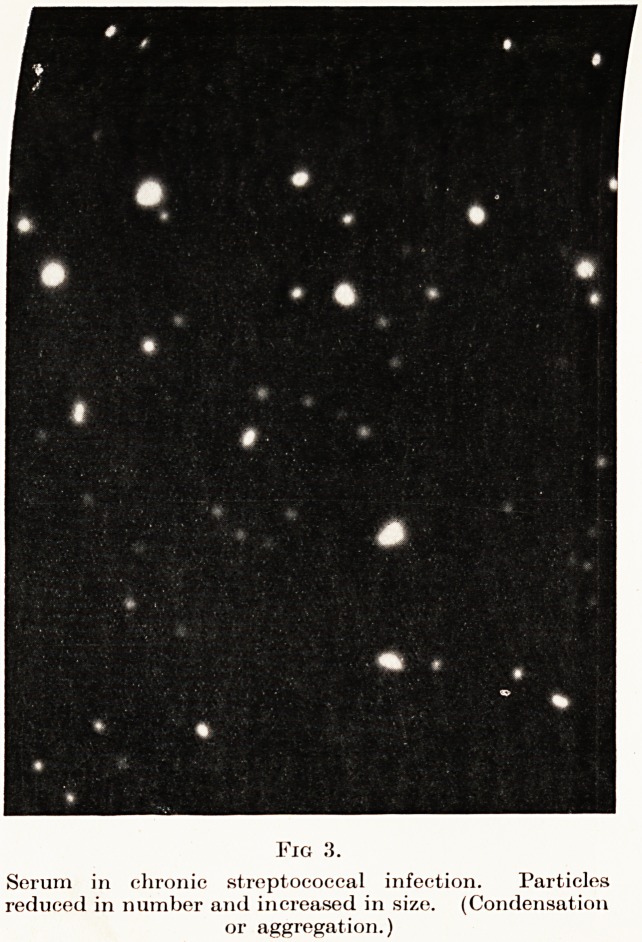


**Fig. 4. f4:**
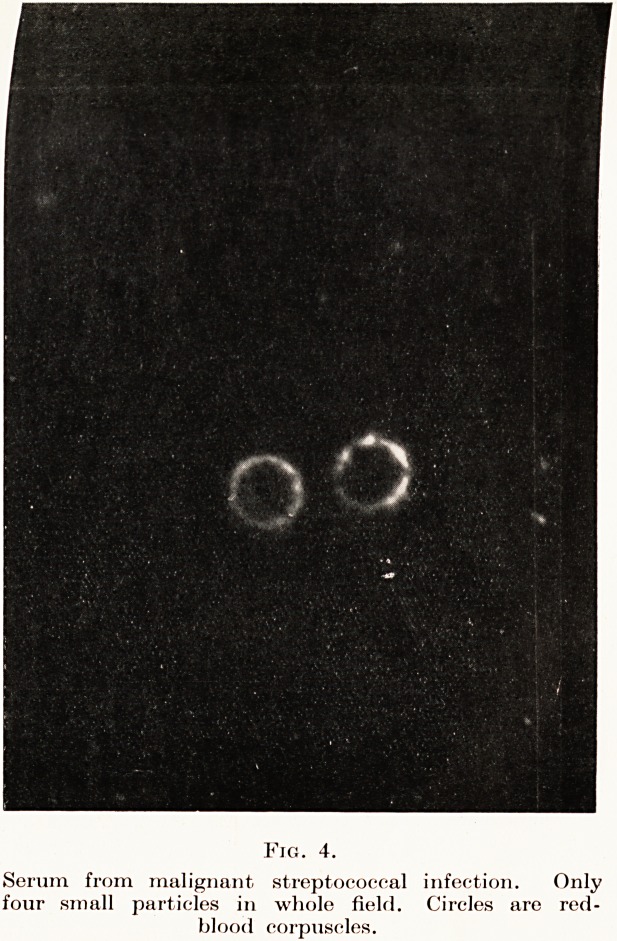


**Fig. 5. f5:**
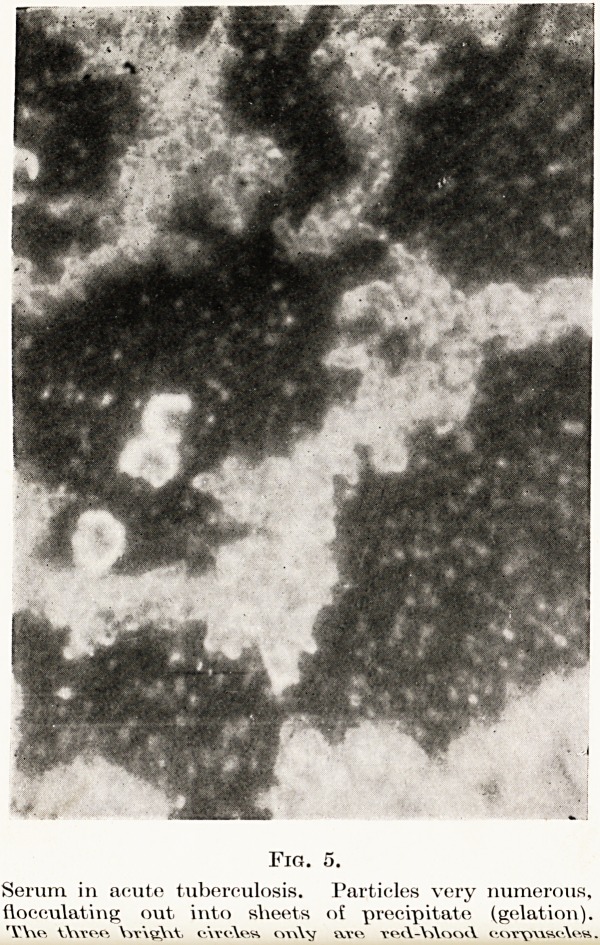


**Fig. 6. f6:**
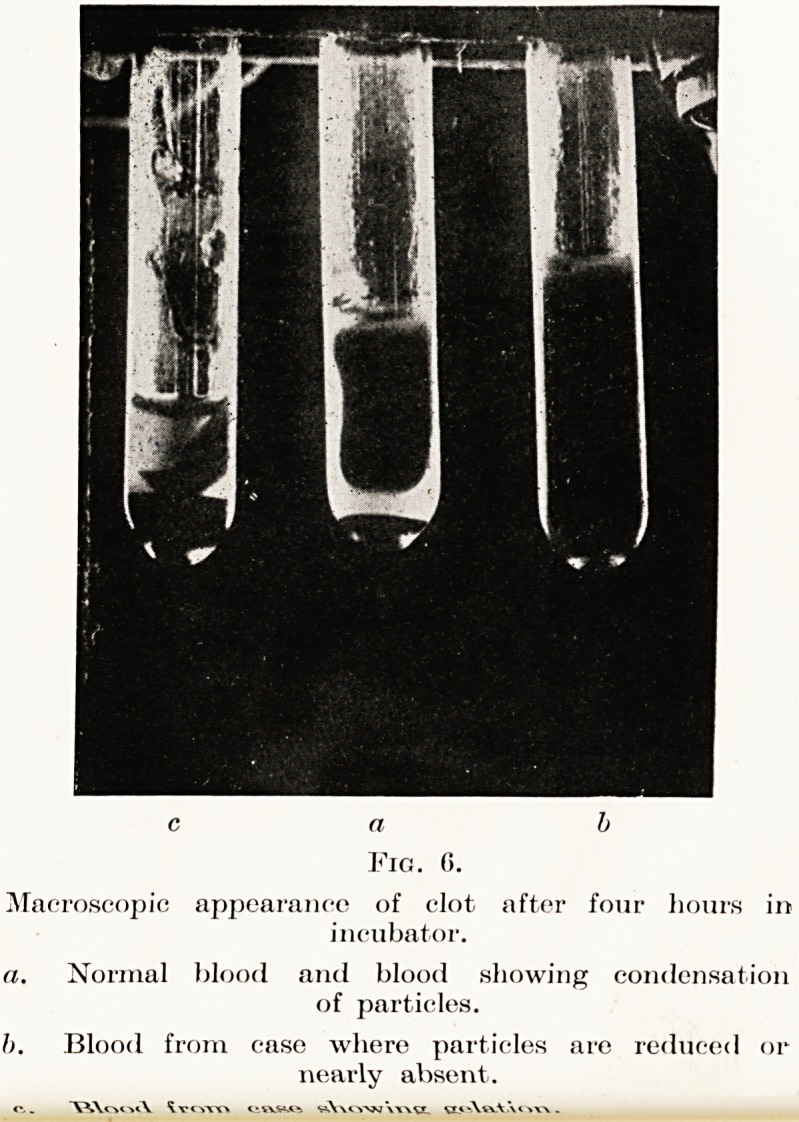


**Fig. 7. f7:**